# Case Report: A rare cause of neonatal respiratory distress: extralobar pulmonary sequestration

**DOI:** 10.3389/fped.2026.1873325

**Published:** 2026-07-29

**Authors:** Natalia Denisiewicz, Lidia Ziętek, Justyna Czubilińska-Łada, Jakub Behrendt

**Affiliations:** Department of Neonatal Intensive Care and Neonatal Pathology, Faculty of Medical Sciences in Zabrze, Medical University of Silesia, Katowice, Poland

**Keywords:** aberrant systematic artery, congenital lung abnormalities, extralobar pulmonary sequestration (ELS), neonatal respiratory distress, sequestrectomy

## Abstract

Pulmonary sequestration (PS) is a rare congenital malformation characterized by a mass of non-functioning, dysplastic lung tissue that lacks normal tracheobronchial communication and receives its blood supply from an aberrant systemic artery. Although advances in prenatal ultrasonography have significantly improved early detection rates, a subset of cases remains undiagnosed until postnatal clinical symptoms emerge. Extralobar sequestration (ELS) in particular typically presents during the neonatal period, frequently manifesting as acute respiratory distress. We present the case of a term male neonate who developed progressive respiratory failure within the first hours of life, despite a completely unremarkable prenatal ultrasound history. Initially managed with non-invasive positive airway pressure, his clinical condition rapidly deteriorated, necessitating endotracheal intubation and invasive mechanical ventilation. Concurrently, the patient was diagnosed with severe congenital pneumonia caused by extended-spectrum *β*-lactamase (ESBL)-producing Klebsiella pneumoniae. While early chest radiographs showed bilateral diffuse opacities without definitive structural anomalies, follow-up imaging on the fifth day of life revealed a large, persistent consolidation in the right lower lobe. Subsequent contrast-enhanced computed tomography angiography (CTA) confirmed the presence of an extralobar pulmonary sequestration measuring approximately 37.5 × 20 × 53 mm. Notably, the CTA identified a rare anatomical variant: the aberrant feeding artery originated directly from the right renal artery, rather than the more typical thoracic or abdominal aorta. Following targeted antibiotic therapy and stabilization of his respiratory status, the patient underwent successful surgical resection of the sequestered lobe on the tenth day of life. Histopathological examination definitively confirmed the ELS diagnosis. The neonate was successfully extubated on the day of surgery, demonstrated an excellent postoperative recovery without requiring supplemental oxygen, and was discharged home on day 22. This case emphasizes the critical need to include congenital lung malformations in the differential diagnosis of unexplained neonatal respiratory failure, even in the context of normal prenatal imaging. Furthermore, the presence of an atypical feeding artery originating from the renal artery underscores the absolute necessity of detailed preoperative vascular mapping with CTA to optimize surgical planning, minimize intraoperative risks, and ensure a favorable clinical outcome.

## Introduction

Pulmonary sequestration (PS) is a rare congenital lung malformation that accounts for approximately 0.15% to 6.40% of all congenital pulmonary anomalies (1, 2). This condition is characterized by a fragment of non-functioning lung tissue that lacks normal communication with the tracheobronchial tree and receives its arterial blood supply from an aberrant systemic artery, most commonly originating from the aorta. The sequestered tissue comprises dysplastic pulmonary structures and can present with a wide spectrum of clinical manifestations (3). In clinical and radiological practice, this entity is often referred to as “bronchopulmonary sequestration” (4).

Pulmonary sequestration is classified into two distinct types based on its pleural investment: extralobar sequestration (ELS) and intralobar sequestration (ILS) (1, 3, 5). The key differences between ELS and ILS are summarized in Table 1 (2). Rare cases involving the coexistence of both forms in a single patient have also been reported in the literature (1, 6). During the prenatal period, PS can be detected as early as the 18th to 19th week of gestation using Doppler ultrasonography (1, 7–9).

**Table 1:** Comparative characteristics of intralobar sequestration (ILS) and extralobar sequestration (ELS).

Feature	Intralobar Sequestration (ILS)	Extralobar Sequestration (ELS)
Prevalence	75%–86%	14%–25%
Location	Contained within the normal lung lobe	Separate from the normal lung lobe
Pleural covering	Shared with the adjacent normal lung	Enclosed in its own visceral pleura
Arterial supply	Most commonly the thoracic aorta	Most commonly the abdominal aorta
Onset of symptoms	Older children and adults	Neonates and infants

In symptomatic infants, particularly those presenting with recurrent respiratory infections or respiratory failure, chest radiography is typically the initial imaging modality and may demonstrate structural abnormalities within the lung parenchyma (3, 10). However, a definitive diagnosis requires detailed imaging to precisely identify the arterial supply and venous drainage of the sequestered segment. Computed tomography angiography (CTA) and magnetic resonance angiography (MRA) are considered the most reliable modalities for evaluating this abnormal vascularization (11–13). On CT imaging, PS may present as a heterogeneous mass containing cystic components or as a fluid-filled lesion (3). Notably, lesions identified during the prenatal period may undergo spontaneous regression or, in some cases, resolve completely (1).

The treatment of choice for clinically symptomatic sequestration is surgical resection, which ensures the complete removal of the affected tissue and prevents complications such as recurrent infections or secondary cyst formation (2). In most cases, pulmonary lobectomy is the preferred surgical approach (1). An alternative or adjunctive treatment strategy is percutaneous embolization of the aberrant feeding artery. This procedure aims to induce ischemia, leading to necrosis and the subsequent involution of the sequestered tissue. When performed preoperatively, embolization can reduce lesion size and decrease the risk of intraoperative bleeding, thereby potentially improving surgical outcomes (14, 15).

## Case presentation

A male neonate from a second pregnancy was delivered at 39 weeks of gestation via cesarean section with a birth weight of 3,890 g and Apgar scores of 8, 9, and 9 at 1, 5, and 10 min, respectively. At 6 h of life, he was transferred to the Neonatal Intensive Care Unit (NICU) due to progressive respiratory distress. Immediately after birth, the neonate demonstrated normal respiratory function and was placed in skin-to-skin contact with his father. However, after several minutes, signs of progressive respiratory distress emerged. The patient was subsequently placed in a medical incubator, and supplemental oxygen therapy was initiated.

As respiratory insufficiency continued to worsen, non-invasive nasal continuous positive airway pressure (nCPAP) ventilation was commenced. Empirical antibiotic therapy with ampicillin and gentamicin was initiated, and the patient was subsequently transferred to the Neonatal Pathology Unit for further evaluation. The maternal medical history revealed a pregnancy complicated by a urinary tract infection during the third trimester. No abnormalities of the fetal respiratory system had been detected during routine prenatal examinations.

Upon admission, the patient was in fair general condition, presenting with pale pink skin and a grayish discoloration, particularly in the thoracolumbar region. Signs of respiratory distress were evident, including tachypnea and intercostal retractions. Auscultation revealed symmetrical vesicular breath sounds over both lung fields. Respiratory support was continued via nCPAP at an FiO2 of 0.3 and pressure settings of 15/5 mmHg. The heart rate was regular at 140 beats per minute, and a cardiac murmur was auscultated.

Laboratory investigations demonstrated a C-reactive protein level within the normal range (0.62 mg/L) but an elevated procalcitonin level above the age-specific reference range (3.99 ng/mL). Additionally, mild elevations were noted in lactate (3.47 mmol/L), aspartate aminotransferase (AST) (75.7 U/L), D-dimer (1.16 μg/mL), and creatine kinase (442 U/L). Capillary blood gas analysis revealed mixed acidosis with the following values: pH 7.28, pCO2 46 mmHg, HCO3 20.6 mmol/L, and a base excess of -6.

**Figure 1:**
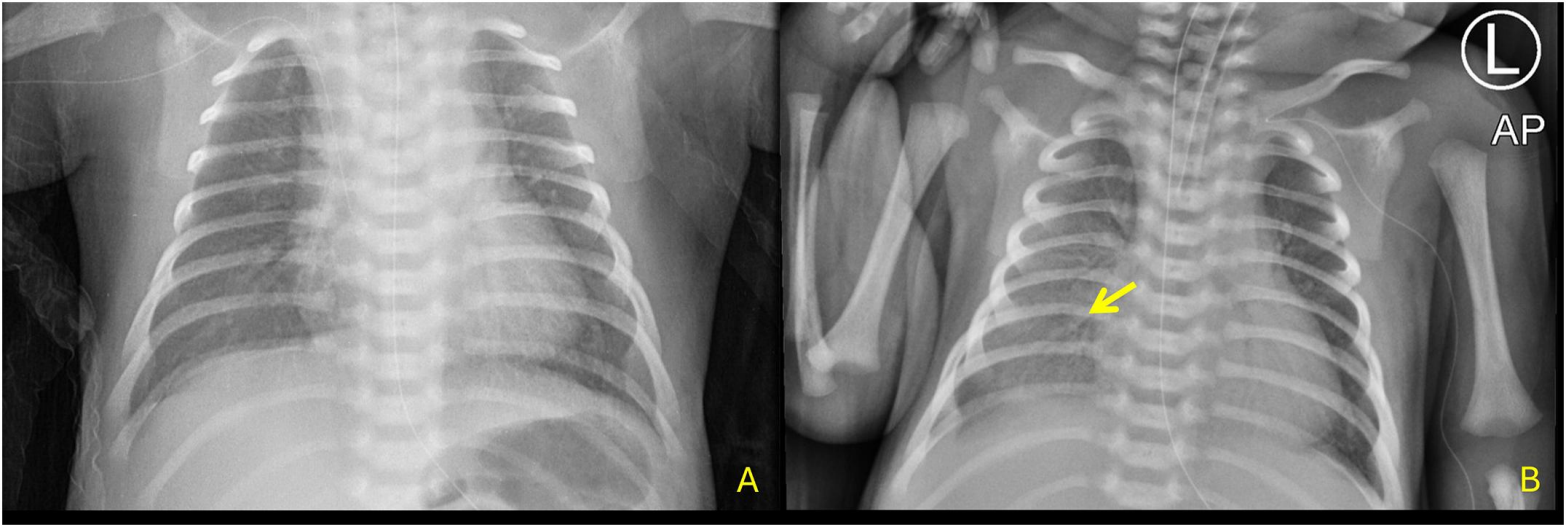
Radiographic progression of the pulmonary sequestration and associated pneumonic changes. **(A)** Anteroposterior chest radiograph obtained on the first day of life. The image demonstrates bilateral diffuse opacities along the bronchovascular bundles without distinct focal mass lesions or definitive features of pulmonary sequestration. **(B)** Follow-up anteroposterior chest radiograph on the fifth day of life, showing significant clinical and radiological progression. A large opacity corresponding to the pulmonary sequestration and extensive consolidative changes is visible in the right lung field (indicated by the yellow arrow), with visible air bronchograms.

A chest ultrasound was performed, demonstrating normal lung contours. Lung mobility during respiration was preserved, and pleural sliding was present throughout the examined pleural surfaces. The anterior lung fields were dominated by A-line artifacts, whereas the posterior lung fields demonstrated B-line artifacts, particularly in the basal regions. Notably, a solid mass measuring measuring 4.1 × 1.6 × 3.9 cm (SD × AP × CC) with distinct arterial vascularization, likely originating from the abdominal aorta, was identified in the right lung, prompting further diagnostic evaluation (Figure 1A).

**Figure 2:**
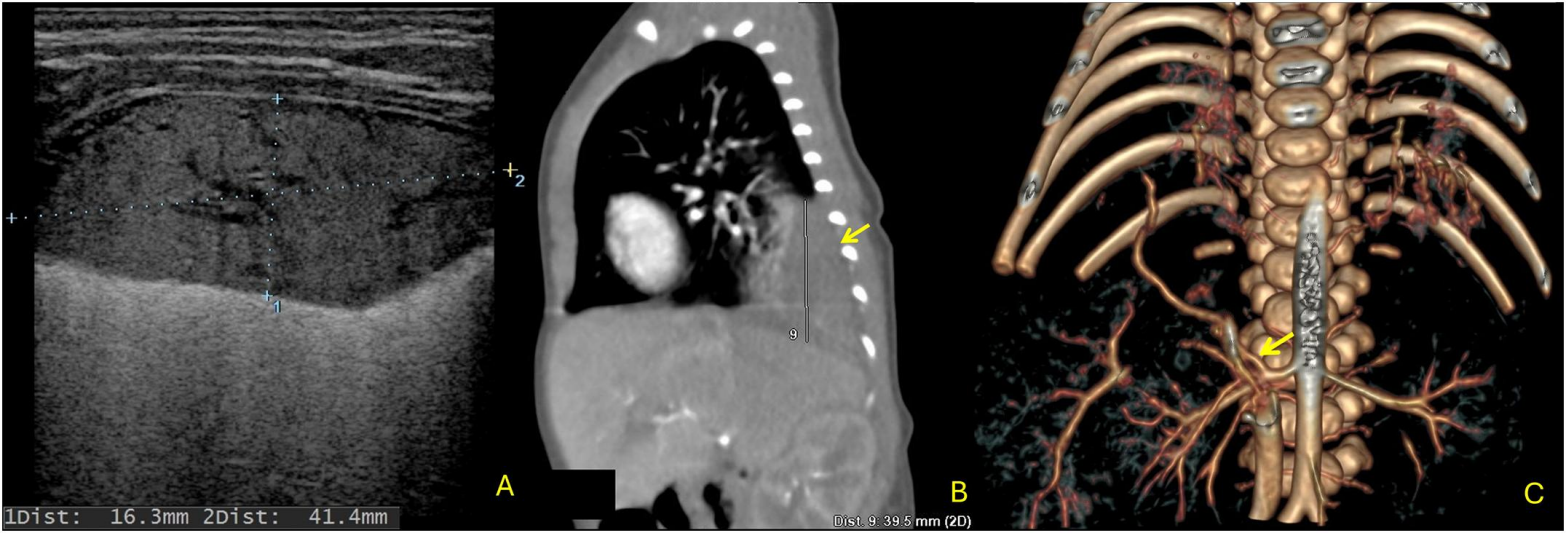
Advanced imaging of the right-sided extralobar pulmonary sequestration. **(A)** Transthoracic chest ultrasound performed on the first day of life. The image demonstrates a well-defined solid mass measuring 4.1 × 1.6 × 3.9 cm (SD×AP×CC) located in the basal regions of the right lung. **(B)** Sagittal view of the contrast-enhanced computed tomography (CECT) on the fifth day of life, revealing a large consolidation measuring 3.8 × 2.0 × 5.3 cm (SD×AP×CC) in the lower lobe of the right lung (indicated by the yellow arrow), accompanied by adjacent atelectatic changes. **(C)** Three-dimensional (3D) CT angiography reconstruction of the vascular supply. The reconstruction clearly delineates the tortuous aberrant systemic feeding artery arising directly from the right renal artery (indicated by the yellow arrow), confirming the diagnosis.

Transcranial ultrasound revealed features consistent with striatothalamic vasculopathy. Due to these abnormal findings and the maternal history of a third-trimester infection, additional diagnostic tests were performed to exclude a TORCH infection. On the day of admission, a chest x-ray revealed bilateral diffuse opacities along the bronchovascular bundles. At this stage, the findings did not indicate the presence of a sequestration-like lesion (Figure 2A).

The radiograph was repeated on the fifth day of hospitalization, demonstrating a large pulmonary sequestration located in the lower lobe of the right lung, spanning approximately four intercostal spaces (Figure 2B). In addition, there was a progression of extensive consolidations involving nearly the entire right lung field—least pronounced at the lung apex—along with visible air bronchograms. The left lung field showed no radiographic abnormalities.

During hospitalization, the patient's respiratory failure progressively worsened. Initially, respiratory support was provided using conventional mechanical ventilation (CMV) and high-flow nasal cannula (HFNC) therapy. However, due to further clinical deterioration on the fourth day of life, mechanical ventilation via synchronized intermittent mandatory ventilation (SIMV) was initiated. Based on the results of bronchial aspirate cultures, the patient was diagnosed with congenital pneumonia caused by extended-spectrum *β*-lactamase (ESBL)-producing *Klebsiella pneumoniae*.

On the fifth day of life, following a surgical consultation, a contrast-enhanced computed tomography (CECT) scan of the chest and abdomen was performed. Imaging revealed an area of consolidation in the lower lobe of the right lung with features consistent with pulmonary sequestration, measuring approximately 3.8 × 2.0 × 5.3 cm (SD × AP × CC) (Figure 1B). The lesion was supplied by an arterial vessel 1.3 to 1.6 mm in diameter, which branched from the right renal artery approximately 6 mm from its origin (Figure 1C). No venous drainage from the sequestrum to the pulmonary veins was visualized, strongly suggesting an extralobar type of pulmonary sequestration. Additionally, atelectatic changes and inflammatory infiltrates were observed in the lower lobe of the right lung.

On the 10th day of life, following the resolution of the pneumonia and a notable improvement in respiratory function, surgical resection of the pulmonary sequestration was performed. The intraoperative course was uneventful. Both the intraoperative assessment and the subsequent histopathological examination confirmed the diagnosis of extralobar pulmonary sequestration. The patient was transferred back to the ward in a serious condition, intubated, and mechanically ventilated in SIMV mode. Extubation, performed later that same day, was successful, and the patient maintained spontaneous breathing without the need for supplemental oxygen.

Follow-up chest radiography revealed a segmental right-sided pneumothorax with a pleural separation of 1 to 2.5 mm. The right lung field showed reduced aeration without any evidence of volume loss. Mild subcutaneous emphysema of the right chest wall was also observed. A chest ultrasound performed on the right side in the postoperative area revealed a trace amount of pleural fluid, with a maximum pleural separation of up to 2 mm. Numerous B-lines and an alveolar pattern were observed in the posterior portion of the right lung.

During the remainder of the hospitalization, the patient remained in good general condition, hemodynamically stable, and with normal respiratory function. After 22 days of treatment, the patient was discharged home and remains under the care of the hospital's neonatal outpatient clinic. A comprehensive timeline summarizing the clinical course, diagnostic work-up, and management is provided in Table 2.

**Table 2:** Timeline of the clinical course and management.

Day of Life/Hospitalization	Clinical Status andInterventions	Diagnostic Evaluations
Day 1 (Birth)	Delivery at 39 hbd via CC; Apgar 8/9/9; progressive respiratory distress at 6 h of life; transfer to NICU. Initiation of nCPAP, empirical antibiotics (ampicillin + gentamicin).	Chest ultrasound: 4.1 × 1.6 × 3.9 cm (SD × AP × CC) mass in the right lung with systemic arterial supply. Initial Chest x-ray: bilateral diffuse opacities, no sequestration-like lesion.
Day 4	Clinical deterioration, worsening respiratory failure; escalation to mechanical ventilation (SIMV).	Bronchial aspirate culture matches: *Klebsiella pneumoniae* (ESBL+).
Day 5	Patient stabilized on mechanical ventilation; surgical consultation.	Repeat Chest x-ray: large pulmonary sequestration in the right lower lobe, extensive consolidations. Contrast-Enhanced CT (CECT): 3.8 × 2.0 × 5.3 cm (SD × AP × CC) mass supplied by an aberrant artery from the right renal artery.
Day 10	Resolution of pneumonia, normalization of inflammatory markers; successful surgical resection of the sequestration; extubation to spontaneous breathing on the same day.	Histopathological examination: confirmed extralobar pulmonary sequestration. Postoperative Chest x-ray & US: mild right-sided pneumothorax, trace pleural fluid.
Day 22	Good general condition, hemodynamically stable, normal respiratory function; discharged home.	Clinical discharge assessment; referred to neonatal outpatient clinic.

## Discussion

Pulmonary sequestration is a rare congenital malformation of the lungs with a heterogeneous clinical presentation that depends primarily on the size and location of the lesion, as well as its impact on the surrounding lung parenchyma. In clinical practice, the extralobar form is of particular importance, as it occurs more frequently during the neonatal or infant period and may present with symptoms of respiratory failure within the first few days of life (1, 16, 17). In contrast to intralobar sequestration, which is often diagnosed later in life due to recurrent respiratory infections, the extralobar form is frequently detected during prenatal imaging or shortly after birth as a result of emerging clinical symptoms.

Advances in prenatal ultrasonography have significantly improved the detection rates of congenital lung malformations. Specifically, Doppler ultrasonography enables the visualization of an abnormal systemic arterial vessel supplying the lesion, which constitutes one of the key diagnostic features of PS (18). Nevertheless, not all lesions are detected prenatally. A small lesion size, unusual anatomical location, or technical limitations inherent to prenatal imaging may result in the defect being overlooked (3, 14). In the present case, no abnormalities of the fetal respiratory system were identified during prenatal ultrasonography. However, the condition became clinically evident within the first few hours after birth, manifesting as progressive respiratory failure. Non-invasive respiratory support was initially applied, but a worsening clinical status necessitated the initiation of mechanical ventilation. Imaging studies showed extensive consolidation involving a significant portion of the right lung, which likely contributed to the patient's severe clinical course.

Postnatal imaging plays a pivotal role in confirming the diagnosis and accurately assessing the vascular anatomy of a pulmonary sequestration. While chest radiography may reveal abnormal opacities or areas of consolidation within the lung parenchyma, it does not allow for precise visualization of the feeding artery. Currently, computed tomography angiography (CTA) is considered the most reliable diagnostic method, as it enables an accurate assessment of both the systemic arterial supply and venous drainage (3, 19).

In the present case, CECT revealed a sequestration lesion supplied by an artery originating from the right renal artery. The most commonly reported source of arterial supply is the thoracic or abdominal aorta; however, less common variants in which the feeding artery arises from other abdominal vessels have also been described in the literature (20–22). The presence of a feeding artery originating from the renal artery represents a rare anatomical variant and highlights the critical importance of a detailed vascular assessment in patients with suspected pulmonary sequestration. Accurate identification of the feeding artery is crucial for appropriate surgical planning and for minimizing the risk of intraoperative complications.

Minor discrepancies between the dimensions of the sequestration mass obtained via ultrasonography 4.1 × 1.6 × 3.9 cm (SD × AP × CC) and computed tomography 3.8 × 2.0 × 5.3 cm (SD × AP × CC) are frequently observed in neonatal practice. These differences are primarily attributable to the intrinsic technical variations between dynamic, operator-dependent acoustic shadowing in transthoracic ultrasound and the static, high-resolution volumetric cross-sectional acquisition of contrast-enhanced CT, as well as shifts in the patient's inspiratory volume and positioning during the respective examinations.

The management of pulmonary sequestration depends primarily on the presence of clinical symptoms. In symptomatic patients, surgical resection remains the treatment of choice, as it allows for the complete removal of abnormal tissue and prevents potential complications such as recurrent pulmonary infections, respiratory failure, or hemorrhage (3). In newborns presenting with respiratory failure, surgical intervention is typically performed only after the stabilization of the patient's clinical condition. In the present case, resection of the lesion was carried out on the 10th day of life, following a marked improvement in respiratory parameters and the normalization of inflammatory markers.

Compared with other cases reported in the literature, the present patient exhibits several significant clinical and anatomical features. First, the lesion was not diagnosed during prenatal imaging, despite the increasing sensitivity of modern ultrasonography. Although an increasing number of PS cases are currently identified prenatally, the literature still reports patients in whom the diagnosis is established only after birth, most often following the onset of clinical symptoms (1). In this case, the disease course was characterized by early and severe respiratory failure requiring mechanical ventilation, a presentation rarely described in newborns. Additionally, the atypical vascular supply of the lesion is noteworthy. The feeding artery arose from the right renal artery, representing a rare anatomical variant. In most reported cases, the arterial supply arises directly from the thoracic or abdominal aorta (3, 20).

In the differential diagnosis of congenital thoracic anomalies presenting in the neonatal period, several entities must be carefully considered, including congenital pulmonary airway malformation (CPAM), hybrid lesions, and congenital lobar overinflation (emphysema). CPAM typically presents as a multicystic mass communicating with the tracheobronchial tree, contrasting with the non-communicating solid nature of extralobar PS. However, hybrid lesions, which share histopathological features of both CPAM and PS, represent a complex clinical challenge; they often manifest with anomalous systemic arterial supply alongside cystic adenomatoid changes. Congenital lobar emphysema, characterized by hyperinflation of one or more pulmonary lobes with normal vascular anatomy, can be distinguished from PS by its hypertranslucent appearance on chest radiographs and the absence of a distinct feeding systemic artery on Doppler ultrasound or CTA (21).

In summary, the present case highlights the importance of considering congenital lung malformations in the differential diagnosis of newborns presenting with respiratory failure. Despite advances in prenatal ultrasonography, pulmonary sequestration may remain underdetected before birth. In such cases, prompt postnatal imaging is crucial, with CTA providing the most accurate assessment of the lesion's vascular anatomy. Early surgical resection following the stabilization of the patient's condition enables definitive removal of the lesion and is associated with a favorable prognosis.

## Conclusion

This case highlights several important clinical considerations in neonatal pulmonary sequestration. First, congenital lung malformations should be included in the differential diagnosis of newborns presenting with respiratory failure, even when prenatal imaging is unremarkable. Second, rare anatomical variants of the feeding artery, such as an origin from the renal artery, underscore the importance of a detailed vascular assessment using CTA. Finally, early surgical resection following the stabilization of the patient is effective and associated with a favorable outcome, emphasizing the value of prompt diagnosis and timely intervention.

## Data Availability

The original contributions presented in the study are included in the article/Supplementary Material, further inquiries can be directed to the corresponding author.
